# Extracellular Traps in Patients Diagnosed With Bacterial Vaginosis, Trichomoniasis, Candidiasis, Noninfectious Vaginitis and Cytolytic Vaginosis

**DOI:** 10.1155/2024/7619416

**Published:** 2024-10-23

**Authors:** María G. Ramírez-Ledesma, Berenice Bermudes-Valencia, Rosa M. Balderas-Parada, Susana G. Salazar-Ramírez, Ruth Reyes-Cortés, Francisco J. Magos-Vázquez, José J. Torres-Hernández, Eva E. Avila

**Affiliations:** ^1^Departamento de Biología, DCNE, Universidad de Guanajuato, Guanajuato, PC 36050, Mexico; ^2^Departamento de Neurobiología Celular y Molecular, Instituto de Neurobiología, Universidad Nacional Autónoma de México (UNAM), Boulevard Juriquilla #3001, Queretaro, PC 76230, Mexico; ^3^Hospital General de Guanajuato Dr. Valentín Gracia, Secretaría de Salud de Guanajuato, Gto-Silao Highway Km 6.5, Guanajuato, Mexico

## Abstract

Vaginal infections are a public health problem associated with serious health complications due to the exacerbated inflammation they generate. Vaginal inflammation may also occur in some noninfectious processes, such as noninfectious vaginitis and cytolytic vaginosis. Immune system cells respond to infections through various mechanisms, such as the formation of extracellular traps (ETs), which are DNA networks associated with effector proteins. Many pathogens induce ETs formation *in vitro*, as occurs in some natural infections. A recent report indicates that human vaginal infections *in vivo* generate ETs. Therefore, in this study, we aimed to identify ETs in samples from 40 donors who were diagnosed with infectious (i.e., bacterial vaginosis, candidiasis and trichomoniasis) and noninfectious (i.e., noninfectious vaginitis and cytolytic vaginosis) vaginal inflammation. We were able to observe ETs by identifying the LL-37 peptide, which is associated with DNA networks. In seven vaginal swabs from the control group (formed by 19 donors without vaginal infection symptoms), we detected at least one pathogen per sample and observed ETs; thus, these donors were considered asymptomatic. The remaining 12 donors were confirmed to be healthy, as their exudates did not present any tested pathogens, sign of inflammation or ETs. ETs in vaginal inflammatory processes can worsen inflammation but may also help control infection.

## 1. Introduction

The healthy human vagina is colonized by a wide diversity of microorganisms, mainly *Lactobacillus* sp. [1, 2], whose population changes with infection. The most common types of vaginal infections are bacterial vaginosis, candidiasis, sexually transmitted diseases like trichomoniasis, and viral infections [1, 3]. During infection, other bacteria, mainly anaerobic species, such as *Gardnerella vaginalis*, *Mycoplasma* sp., *Escherichia coli* and *Staphylococcus aureus,* replace the normal vaginal microbiota. Bacterial vaginosis generates profuse vaginal flow with a high pH (> 5) and an unpleasant odour [4].

Opportunistic fungi of the genus *Candida* cause candidiasis, which is characterised by profuse, white, clumpy vaginal discharge [5, 6]. Trichomoniasis, caused by the parasite *Trichomonas vaginalis*, generates excessive inflammation in the genital tract, copious yellowish vaginal exudate with a high pH (> 5), and a decrease in the normal microbiota [6, 7, 8].

Noninfectious inflammatory processes, such as cytolytic vaginosis and noninfectious vaginitis, also occur in human vaginal epithelium. Cytolytic vaginosis is characterised by overgrowth of normal *Lactobacillus* sp. microbiota, increased vaginal discharge, decreased pH and absence of pathogens [9, 10]. In noninfectious vaginitis, the vaginal epithelium becomes inflamed or irritated because of various causes such as hormonal changes, menses, vaginal douching, sexual practice and uncontrolled usage of antibiotics [11].

Vaginal infections are a public health issue that is associated with severe health complications, such as pregnancy loss, premature births, low-weight newborns [12], infertility [13] and increased susceptibility to sexually transmitted diseases [14]. These complications result from the exacerbated inflammation that occurs during infection.

Neutrophils and macrophages are cells of the innate immune system that defend the female genital tract [15, 16, 17] and contribute to the inflammatory process. These cells, along with eosinophils, mast cells, and basophils, use several defence mechanisms, such as the formation of extracellular traps (ETs) [18, 19]. ETs are generated *in vitro* and *in vivo* in natural infections, including those of the genito-urinary tract [20]. Immune cells form and release these DNA networks in response to pathogens—or their components—and certain immune molecules, even in noninfectious inflammatory diseases [21, 22]. ETs can kill microorganisms and prevent them from spreading [23].

This study aimed to identify ETs in exudates from donors with symptoms of vaginal infection or inflammation. Our results indicate the presence of ETs in donors with bacterial vaginosis, candidiasis, trichomoniasis, noninfectious vaginitis and cytolytic vaginosis.

## 2. Materials and Methods

### 2.1. Ethics Statements

Vaginal exudate samples were obtained from 59 female voluntary donors. All donors were informed about the study according to the Helsinki protocol. The inclusion criteria for healthy donors (Group 1, 19 donors) were as follows: age > 18 years, not pregnant, not diagnosed as a human immunodeficiency virus (HIV) carrier, no symptoms of infection or inflammation, and visiting the laboratory only for a Papanikolaou test. The inclusion criteria for patients (Group 2, 40 donors) were the same as those for healthy donors, except this group did report symptoms of vaginal infection or inflammation and their physicians had indicated laboratory tests of vaginal exudates. The exclusion criteria were pregnant women and patients diagnosed as HIV positive.

The samples were obtained at the Dr. Valentín Gracia General Hospital, and the diagnosis was made by following the usual procedures for all patients at the Clinical Laboratory of the same hospital. The presence of ETs was analysed at the Biology Department of the DCNE of the Universidad de Guanajuato. The Institutional Bioethics Committee of the Universidad de Guanajuato (Code: CIBIUG-P042-2015) and the Research Ethics Committee of Secretaría de Salud de Guanajuato (Code: CEI-HGTO2016-001) approved the protocol. Sample remnants were discarded in accordance with Mexican regulations (NOM-087-ECOL-SSA1-2002, NOM-007-SSA3-2011). We analysed the laboratory results of “healthy” donors to use them as negative controls, meaning that the donors were asymptomatic, and their samples contained no detectable pathogens.

### 2.2. Patient Data Record

We requested patient data such as age, pregnancy, general health status and concomitant diseases. Likewise, we registered vaginal infection symptoms and the names and doses of current prescribed and nonprescribed medications, antimicrobials, anti-inflammatories, immunosuppressants, vitamins and others. In addition, donors filled out and signed an informed consent form.

### 2.3. Clinical Diagnosis and Pathogen Identification

Vaginal discharge was assessed by quantity, consistency, colour and pH. The laboratory at Dr. Valentín Gracia General Hospital provided the clinical diagnosis and identified the pathogens using the MicroScan Walk-Away Plus System 96, an automated equipment for bacterial biochemical test panels. We microscopically analysed the fresh samples to identify desquamative cells, leucocytes, yeasts and parasites. Gram staining was used to determine bacterial morphotypes and key cells and Nugent and Amsel criteria were used for results interpretation [24].

### 2.4. Sample Processing

On clean and sterile slides pretreated with poly-L-lysine (Sigma-Aldrich, cat. P0425-72EA), we made three smears with the vaginal exudate. The samples were fixed with 4% paraformaldehyde at room temperature for 15 min and transported to the research laboratory for treatment. Slides were washed with phosphate-buffered saline (PBS) to remove excess paraformaldehyde.

### 2.5. Gram Staining

Slides (a) were incubated with Crystal violet (Hycel, cat. 680) for 1 min and washed with distilled water. Iodine solution (Hycel, cat. 2688) was applied for 1 min. Next, slides were washed with distilled water and then with alcohol-acetone solution for 15 s (2:1, v/v). Then, the slides were incubated with Safranin solution (Hycel, cat. 826) for 1 min, washed with distilled water, air-dried and observed under an optical microscope.

### 2.6. Immunodetection of LL-37 and *T. vaginalis* in Patient Samples

Slides marked (b) and (c) were immersed in 100 mM glycine solution (Promega, cat. H5071) and incubated for 6 h at room temperature. Nonspecific binding was blocked with a solution containing 10% decomplemented human plasma, 0.1% pig gelatin and 0.1% Tween 20 in sterile PBS. For *T. vaginalis* identification, slides marked with the letter (b) were treated with house-made antibodies against the parasite in PBS-0.05% Tween 20 [25], and then with an Alexa Fluor 594-conjugated secondary antibody against rabbit IgG (Invitrogen, cat. A21207). DNA was stained with Hoechst 33342 (Invitrogen, cat. H1399). Prolong Gold (Invitrogen, cat. P36930) was added and samples were observed under a Carl Zeiss, LSM700 confocal microscope.

We used samples marked with (c) for ETs identification by the detection of extracellular DNA associated with LL-37. These samples were incubated with rabbit antibodies against LL-37 (Santa Cruz Biotechnology, cat. 8C-50423) in PBS-0.05% Tween 20 and then with an Alexa Fluor 594-conjugated secondary antibody against rabbit IgG (Invitrogen, cat. A21207). DNA was stained with Hoechst 33342. Afterwards, samples were treated identically to those labelled as (b).

## 3. Results

### 3.1. Negative Control Samples

We did not detect any pathogen in 12 (63%) of the 19 samples from the asymptomatic donors (lacking symptoms of vaginal infection or inflammation, Group 1). Additionally, we reclassified two (5%) of the 40 samples from the symptomatic donors (Group 2) because they did not present any pathogen, resulting in 14 negative control samples. The donors of these samples were between 28 and 58 years old, and their vaginal discharges were of regular abundance, with a homogeneous texture, whitish in colour, and with a pH range of 4.0–4.5. All 14 exudates presented normal *Lactobacillus* spp. dominated microbiota (Gram-positive) and sparse epithelial cells and did not exhibit pathogens or ETs. Four of the 14 donors were under medication: one donor had dyslipidaemia and took pravastatin, another had hypertension regulated by losartan, and two donors had diabetes controlled by insulin. Despite the diseases and medication intake, the characteristics of the vaginal exudates from these patients were like those without concomitant diseases.

**Figure 1:**
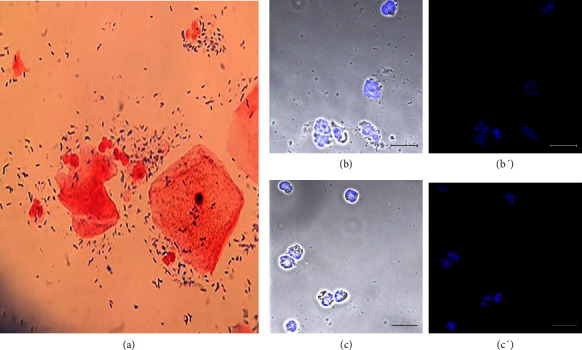
Sample from an asymptomatic donor without vaginal infection or inflammation. Donor number 3 had no symptoms or signs of infection or inflammation. (a) Gram staining showed normal microbiota; (b and b′) negative staining of *T. vaginalis* with specific antibodies and a secondary antibody coupled Alexa Flour 594; (c and c′) multilobed nuclei of neutrophils with Hoechst 33342 dye and LL-37 immunodetected in their cytoplasm, bar 20 μm.

Figure 1 shows the results of healthy donor 3. Gram staining revealed normal microbiota in this sample (Figure 1(a)). The search for *T. vaginalis* using antibodies against the parasite was negative (Figures 1(b) and 1(b′)). Sample 3 did not show ETs, presenting only neutrophils in the normal range (< 10 neutrophils/field) with the characteristic LL-37 staining in their cytoplasm (Figures 1(c) and 1(c′)).  Figure 1s shows the results of healthy donor 51 without infection or inflammation.

### 3.2. Donors With Vaginal Infection

There were 45 donors with vaginal infection: 38 (from the initial 40) symptomatic patients sent to the laboratory by their physicians and with an identified inflammatory condition or pathogen (Group 2); seven asymptomatic persons (from Group 1) that this study found positive for a pathogen or inflammatory condition. Using the diagnoses provided by the laboratory, we classified the 45 samples as noninfectious vaginitis, cytolytic vaginosis, bacterial vaginosis, trichomoniasis and candidiasis.  Table 1 shows the number of samples classified in each group.

### 3.3. Donors Diagnosed With Noninfectious Vaginitis

**Figure 2:**
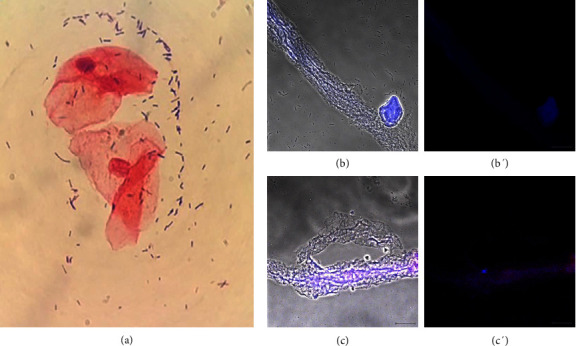
Sample of a patient diagnosed with noninfectious vaginitis. Donor number 10 had symptoms of vaginal infection or inflammation. (a) Gram staining showed Gram-positive bacilli corresponding to the normal microbiota (*Lactobacillus* sp.); (b and b′) the immunodetection of *T. vaginalis* was negative; (c and c′) DNA networks were present (blue, Hoechst 33342) associated with LL-37 (red). Bar 20 μm.

Donors were diagnosed with noninfectious vaginitis if they had vaginal inflammation but no tested pathogens. The 13 donors in this group were 31–60 years old and represented 29% of the 45 donors with a diagnosed vaginal condition. The symptoms they presented were mainly pain, dryness, and itching in the genital area. Donors with this diagnosis had scant–regular vaginal discharge, which as slightly cloudy, white to light yellow, and with a pH of 5.0–8.0. The microbiological and biochemical analysis did not reveal any pathogens. Normal *Lactobacillus* sp. dominated microbiota (Gram-positive) and abundant squamous epithelial cells were observed (Figure 2(a)). *T. vaginalis* immunodetection was negative (Figures 2(b) and 2(b′)). In these samples, we observed extracellular DNA networks (blue) associated with LL-37 (red), evidencing ETs formation (Figures 2(c) and 2(c′)). A 52-year-old donor was undergoing insulin treatment to control type 2 diabetes mellitus, but her sample had similar characteristics to those of the other donors with this diagnosis. Figure 2 came from donor number 10, one of the 13 diagnosed with noninfectious vaginitis.

### 3.4. Donors Diagnosed With Cytolytic Vaginosis

**Figure 3:**
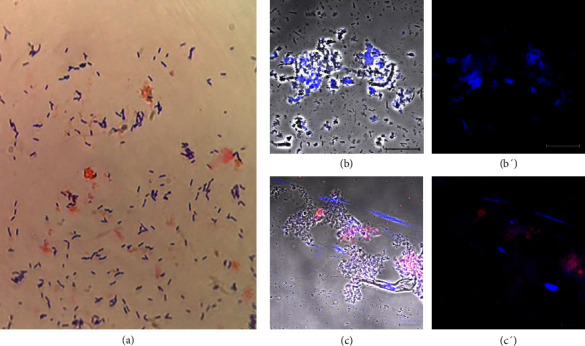
Sample from a donor diagnosed with cytolytic vaginosis. Donor number 6 reported symptoms of vaginal infection or inflammation. (a) Gram staining showed an excessive number of Gram-positive bacilli, *Lactobacillus sp*, but the absence of pathogens; (b and b′) the negative detection of *T. vaginalis;* (c and c′) the presence of extracellular DNA stained with Hoechst 33342 (blue) and anti-LL-37 antibodies (red), bar 20 μm.

Nine (20%) of the 45 donors with a vaginal disorder had cytolytic vaginosis. Their ages ranged from 23 to 51 years, and none of them had comorbidities. They described their main symptoms as pruritus, irritation, pain in the genital area, and increased vaginal discharge, which was regular to abundant, white, and cloudy, and with a pH of 3.5–4.5. Samples showed squamous epithelial cells and a higher number of *Lactobacillus* sp. (> 30 per field) compared to the standard. The samples were without *C. albicans* (Figure 3(a)) or *T. vaginalis* (Figures 3(b) and 3(b′)). All the exudates of this group presented ETs of DNA (Figures 3(c) and 3(c′)). Figure 3 depicts a representative sample (donor 6) of the group with cytolytic vaginosis.

### 3.5. Donors Diagnosed With Bacterial Vaginosis

The group of donors with bacterial vaginosis is made up of nine symptomatic donors (one with concomitant trichomoniasis) from Group 2 and five asymptomatic donors that were reclassified from Group 1. The symptomatic donors were 24–80 years old and did not have concomitant diseases. Their most frequent symptoms were pain, inflammation, irritation and itching in the genital area. Three donors had scant vaginal discharge that was yellow to slightly brown, whereas five donors had regular to abundant, white and cloudy vaginal discharge. The pH was of 5.0–6.0.

The asymptomatic donors comprised five women in the age range of 29–44 years old. Donor 57 (age 44) was under medical treatment with Rivaroxaban for venous insufficiency, and donor 59 (age 41) for arthritis with prednisone. They had abundant, cloudy, white vaginal discharge with a pH from 4.5 to 5.0. The other three donors in the group did not have concomitant diseases.

The clinical laboratory reported that, in the 14 donors, the normal microbiota was replaced by other bacteria. We identified *Escherichia coli* in 10 samples, *Staphylococcus aureus* in three, and *Streptococcus gallolyticus* in one. There was no relationship between the characteristics of the vaginal discharge and the bacteria we found.

**Figure 4:**
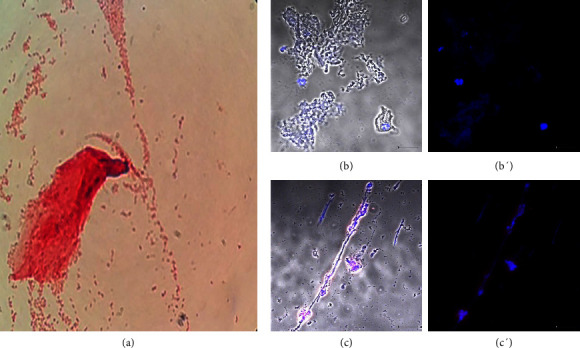
Sample from a donor diagnosed with bacterial vaginosis. Donor number 20 presented symptoms of vaginal infection. (a) Staining showed Gram-negative bacilli and the microbiological analysis growth of *E. coli;* (b and b′) *T. vaginalis* was negative; (c and c′) Hoechst 33342 stain (blue) and LL-37 antibodies (red) showed the presence of extracellular traps. Bar 20 μm.

Figure 4 shows the results of symptomatic donor number 20 with bacterial vaginosis caused by *E. coli*. *S*taining showed Gram-negative bacilli (Figure 4(a)).  Figure 2s shows sample 50 from an asymptomatic donor diagnosed with *Staphylococcus aureus*. The donors in this group did not show the presence of the *T*. *vaginalis* parasite (Figures 2s(b), 2s(b′), 4(b) and 4(b′)). All samples taken from donors with bacterial vaginosis showed ETs (Figures 2s(c), 2s(c′), 4(c) and 4(c′)). Donors with bacterial vaginosis represented 31% of those with a vaginal condition.

### 3.6. Donors Diagnosed With Trichomoniasis

**Figure 5:**
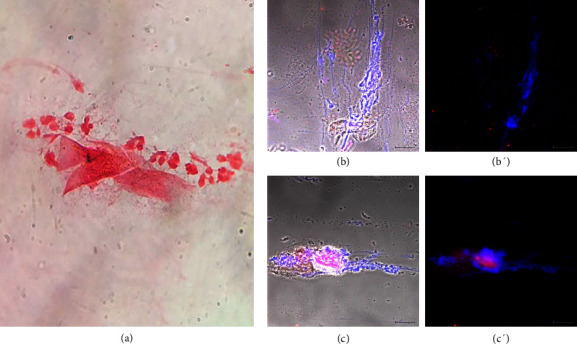
Sample from a donor diagnosed with trichomoniasis. Symptomatic donor number 24 was diagnosed with trichomoniasis. (a) Gram staining showed the absence of *Lactobacillus* sp; (b and b′) specific antibodies *α-T. vaginalis* and a secondary antibody coupled to Alexa Fluor 594 detected the parasite (red); (c and c′) extracellular DNA and multilobed neutrophil nuclei (blue) stained with the Hoechst 33342 dye and antibodies anti-LL-37 (red). Bar 20 μm.

Five donors were diagnosed with trichomoniasis: one asymptomatic and four symptomatic. These donors represented 11% of the 45 donors with a vaginal disorder. The age of the symptomatic donors ranged from 34 to 86 years old. None had been diagnosed with a concomitant disease. The most frequent symptoms they presented were pain and itching. Their vaginal discharge was reported to be abundant, cloudy, and light to intense yellow, with a pH of 5.0–6.0. The Gram stain of symptomatic donor 24 (age 52) revealed only scarce normal microbiota (Figure 5(a)). Asymptomatic donor 48, (age 44) presented abundant, cloudy, and yellow vaginal discharge with a pH of 6.5. The Gram stain shows normal Gram-positive microbiota (Figure 3s(a)). Donor 13 exhibited *T. vaginalis* and abundant bacteria identified as *E. coli* by the MicroScan Walk-Away Plus System 96.

The microscopic analysis of the exudates showed *T*. *vaginalis* trophozoites and some epithelial cells (Figures 3s and 5). We confirmed the presence of *T. vaginalis* in all samples by confocal microscopy and identified entire trophozoites and some debris (red) apparently interacting with DNA networks (Figures 3s(b), 3s(b′), 5(b) and 5(b′)). The DNA of ETs associated with LL-37 (Figures 3s(c), 3s(c′), 5(c) and 5(c′)) confirmed the presence of ETs in samples of this group.

### 3.7. Donors Diagnosed With Candidiasis

**Figure 6:**
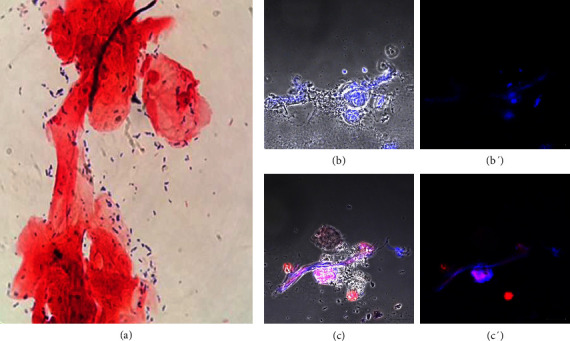
Sample from a donor diagnosed with candidiasis. Donor number 27 presented symptoms of vaginal infection. (a) Gram staining showed normal microbiota, yeasts and pseudohyphae; the microbiological study was diagnosed with candidiasis; (b and b′) the immunodetection of *T. vaginalis* was negative; (c and c′) Extracellular DNA stained with Hoechst 33342 (blue) and LL-37 immunodetected (red). Bar 20 μm.

Five donors were diagnosed with candidiasis, representing 11% of the patients with a diagnosed vaginal condition. Out of the five donors, one was asymptomatic and four were symptomatic. The symptomatic donors (ages 28–41) did not have concomitant diseases; their most frequent complaints were pain, itching, and irritation in the genital area. The vaginal discharge was profuse, white, and lumpy, with a pH of 4.0–5.0. Samples presented yeast, pseudohyphae and normal microbiota in fresh analysis and Gram stain (Figure 6(a)) but no other pathogens (Figures 6(b) and 6(b′)). Samples with *Candida* showed extracellular DNA associated with the antimicrobial peptide LL-37 (Figures 6(c) and 6(c′)). Figure 6 depicts the sample taken from donor 27 (age 30).

Donor 49 (age 28) was asymptomatic. Her vaginal discharge was abundant, white, and lumpy, with a pH of 5.0; it presented yeasts, *Lactobacillus* sp. (Figure 4s(a)), absence *T. vaginalis* (Figures 4s(b) and 4s(b′)) and ETs (Figures 4s(c) and 4s(c′)).

## 4. Discussion

In this study, we observed the presence of ETs in vaginal swab samples taken from women suffering from infectious and inflammatory processes: bacterial vaginosis, trichomoniasis, candidiasis, cytolytic vaginosis and noninfectious vaginitis. ETs occur under natural infectious conditions, such as large amounts of circulating neutrophil ETs (NETs) in septic patients, and under noninfectious conditions in systemic autoimmune diseases like lupus erythematosus [26] and rheumatoid arthritis [27]. Pathogens of the female genital tract *in vitro* [23, 28, 29, 30, 31] and *in vivo* [20] have been reported that induce the ETs formation. Our results are consistent with these reports and demonstrate the presence of ETs when there are no pathogens, similar to noninfectious vaginitis or cytolytic vaginosis. ETs could contribute to the defence and elimination of pathogens, but also to the excessive inflammation generated in the vaginal tract, even lacking an infectious cause.

Our study also supports many scientific reports indicating that ETs are formed in the innate response of microorganisms. Numerous pathogens induce ET formation: bacteria, parasites, fungi, and viruses. These reports were obtained from *in vitro* and *in vivo* experiments, including natural infectious processes and sterile inflammatory conditions.

Therefore, we expected and observed ETs in samples with genito-urinary infections, which agrees with the findings by Zambrano et al., who analysed NET formation in 10 vaginal swabs from patients diagnosed with bacterial vaginosis, candidiasis and trichomoniasis [20]. Our study included samples from 38 symptomatic and 7 asymptomatic donors with bacterial vaginosis, trichomoniasis, candidiasis and inflammatory conditions. Surprisingly, all the samples with inflammatory noninfectious processes, such as cytolytic vaginosis and noninfectious vaginosis, exhibited ETs. Like many immune mechanisms, ETs are a double-edged sword. Several studies suggest or demonstrate that ETs are relevant in pathogen elimination. For example, *T. vaginalis* viability *in vitro* decreases when co-incubated with human NETs as opposed to neutrophils that do not form NETs [28]. The growth of other parasites, including *Plasmodium falciparum* [32] and *Toxoplasma gondii*, is limited by NETs formation [33]. Furthermore, NETs kill bacteria like *E. coli*, *Salmonella* typhimurium, *Klebsiella pneumoniae*, *Pseudomonas aeruginosa* [23] and fungi like *C. albicans* [34]. Conversely, ETs cause serious complications if they are present in the blood circulation, as may occur in systemic lupus erythematosus, rheumatoid arthritis, antineutrophil cytoplasmic antibody–associated vasculitis, antiphospholipid antibody syndrome and idiopathic inflammatory myopathies [27]. Even if ETs are induced in mucosal surfaces, they may contribute to exacerbate the inflammatory process. In conclusion, this study is relevant because it demonstrates that ETs are present widely in infectious and noninfectious processes in vaginal epithelium. More studies are necessary to determine the contribution of ETs in the elimination of genito-urinary pathogens and to inflammation.

Although the precise cellular source of ETs in vaginal swab samples was not shown, they are human immune cells because they stain with an antibody against the antimicrobial peptide LL-37. Neutrophils are good candidates to be the primary source of ETs since these immune cells are the most abundant in vaginal infectious processes, such as trichomoniasis [16, 17], which did not exclude the contribution of other immune cells. Neutrophils, mast cells and monocytes all contain LL-37 [19, 35, 36, 37], which was associated with ETs in this study.

More than 45 species of lactobacilli comprise the vaginal microbiota and build a barrier that limits the growth of pathogens or opportunistic microorganisms, contributing to the protection of the epithelium [38]. The hallmark of cytolytic vaginosis is the excessive growth of lactobacilli. Despite being nonpathogenic, these bacteria damage the epithelium and produce an inflammatory response. The signs and symptoms of cytolytic vaginosis are often confused with those of candidiasis [9]. In a 2004 study of 210 donors with suspected infection, 7.1% were diagnosed with cytolytic vaginosis [39]. In our study, 20% of the 45 donors with a vaginal disorder had cytolytic vaginosis, and none of them had a history of diabetes. This is a relevant finding because scientific reports indicate that the number of lactobacilli increases in women with elevated serum glucose [39]. Cytolytic vaginosis is characterized by few, if any, leucocytes [40]. Consistently, we did not observe leucocytes in the samples of this group. However, we observed ETs. Some noninfectious proinflammatory stimuli may induce the formation of ETs, such as IL-8, TNF-*α* and IFN-*γ*, in these donors [41, 42].

Although there is little research on noninfectious vaginitis, a study published in 2010 reported that this condition is caused by certain types of underwear, as well as allergies or trauma due to excessive tampon use, douches, lubricants, vaginal deodorants and other hygiene products can also cause noninfectious vaginitis. These products generate an inflammatory response without the demonstrable presence of a pathogen [11, 43]. It is unknown if any of these extracorporeal compounds can directly or indirectly induce ETs formation. In our study, 29% of the 45 donors with a vaginal condition were diagnosed with noninfectious vaginitis. We did not find any pathogen in the samples, but all showed ETs, which were possibly induced by proinflammatory compounds.

Lactobacilli are absent in bacterial vaginosis, and there is an overgrowth of anaerobic and facultative bacteria [44]. *Gardnerella vaginalis* species predominate in many bacterial vaginosis samples [45]. Prevalence rates for this infection vary by country but usually range between 20% and 60% [46]. We found that 31% of the 45 donors with a vaginal disorder had bacterial vaginosis, and the more frequent etiological agents were *E. coli* and *S. aureus*. Pathogenic bacteria trigger an inflammatory response that leads to high levels of the proinflammatory cytokines IL-1*β*, IL-6 and IL-8 [47], thereby causing the complications associated with bacterial vaginosis [48]. The samples of these donors showed ETs, which is consistent with previous observations that *in vitroS. aureus* and *E. coli* induce the formation of neutrophil and mast ETs [23, 30, 31]. One of the samples from a donor carrying *Streptococcus gallolyticus* also presented ETs, although there are no reports of ETs formation induced *in vitro* by this pathogen. *S. gallolyticus* is an emerging human pathogen that was likely contaminating the genital area [49]. Regarding symptoms, up to 50% of reproductive-age women with bacterial vaginosis could be asymptomatic [50]. This study showed that, out of the 14 donors with bacterial vaginosis, five (between the ages of 29 and 44) were asymptomatic.

Five donors had trichomoniasis (four symptomatic and one asymptomatic). According to several reports, up to 50% of women with trichomoniasis are asymptomatic [51]. Vaginal exudates from women infected with *T. vaginalis* contain higher concentrations of the proinflammatory cytokines IL-8, IL-22 and IL-17 [52, 53], which may be associated with ETs production. We observed ETs in the five samples of this group. Accordingly, *T. vaginalis* induces the formation of mouse [54] and human [28] NETs *in vitro* and in the murine peritoneum [55]. Zambrano et al. reported the presence of NETs in three vaginal swab samples from women infected with *T. vaginalis*. One of these samples exhibited bacterial vaginosis [20] in addition to trichomoniasis. This is because the establishment of *T. vaginalis* is associated with a decrease in the normal microbiota, thereby contributing to the development of pathogenic bacteria [51].

Candidiasis is one of the most common vaginal infections and may be asymptomatic in more than 50% of the cases. *Candida albicans* causes 75% of vulvovaginal candidiasis, and other *Candida* species cause the remaining 25% [5]. Neutrophils, macrophages and dendritic cells contribute to the innate immune response to *Candida* sp. These cells respond to the different morphotypes of *Candida* (pseudohyphae and yeast) through mechanisms such as ET formation [29]. Previous reports have shown that *Candida albicans* triggers NETs *in vitro* [34, 56], and vaginal samples of women with candidiasis have these traps [20]. Mast cells, monocytes and macrophages also induce ETs *in vitro* [19, 57]. This study found that all five donors with candidiasis exhibited ETs; one was asymptomatic.

Two of the 40 symptomatic donors did not have a diagnosed pathogen. The donors reported vaginal discomfort; however, we did not detect any pathogens in their samples. Both donors were menopausal women (ages 50 and 52) whose symptoms are likely explained by hypoestrogenism. Menopausal women often present with vaginal dryness, cell shedding and an altered microbiome [58]. Negative control samples from healthy donors did not exhibit any pathogens, only lactobacilli. A limitation of this study is that we did not identify the cell type that produces ETs in vaginal exudates. Our main finding was that ETs usually arise under natural inflammatory conditions such as noninfectious vaginitis and cytolytic vaginosis and in infectious diseases.

## 5. Conclusion

This study demonstrates that ETs are widely distributed in both infectious and noninfectious processes within the vaginal epithelium. Vaginal exudates from women with and without symptoms of vaginal infections showed that the cells of the immune system naturally form ETs. These DNA traps occur in noninfectious vaginitis and cytolytic vaginosis, as well as in infections caused by pathogens of the female genital tract, such as anaerobic bacteria, *Candida albicans* and the parasite *Trichomonas vaginalis*. ETs were presented similarly in symptomatic and asymptomatic donors.

## Figures and Tables

**Table 1:** Samples from donors with and without symptoms of infection.

	Without infection	Noninfectious vaginitis	Cytolytic vaginosis	Bacterial vaginosis	Trichomoniasis	Candidiasis
Symptomatic donors	2	13	9	9	4	4
Asymptomatic donors	12	—	—	5	1	1
ETs	0	13	9	14	5	5

*Note:* Nineteen initial vaginal exudates were obtained from “healthy” or asymptomatic donors (Group 1) and 40 from symptomatic donors (Group 2). After laboratory diagnosis, 45 donors resulted with an infectious or inflammatory condition; two were symptomatic without any detected pathogen, and 12 were healthy donors without signs of infection or inflammation. One donor presented concomitant trichomoniasis and bacterial vaginosis. Therefore, she was counted in both groups.

## Data Availability

Data are available on request from the authors.

## Supporting Information

Supporting Figure 1: Sample from a clinically healthy donor without infection or inflammation. Supporting Figure 2: Sample from an asymptomatic donor diagnosed with bacterial vaginosis. Supporting Figure 3: Sample from an asymptomatic donor with trichomoniasis. Supporting Figure 4: Sample from an asymptomatic donor with candidiasis.
