# Supplementary material for: Extracellular Traps in Patients Diagnosed With Bacterial Vaginosis, Trichomoniasis, Candidiasis, Noninfectious Vaginitis and Cytolytic Vaginosis
Source: Int J Microbiol. 2024 Oct 23;2024:7619416. doi: 10.1155/2024/7619416 (PMC11524696; doi:10.1155/2024/7619416)
Supplement: Supporting Information — Additional supporting information can be found online in the Supporting Information section. [file 7619416.f1.pdf]

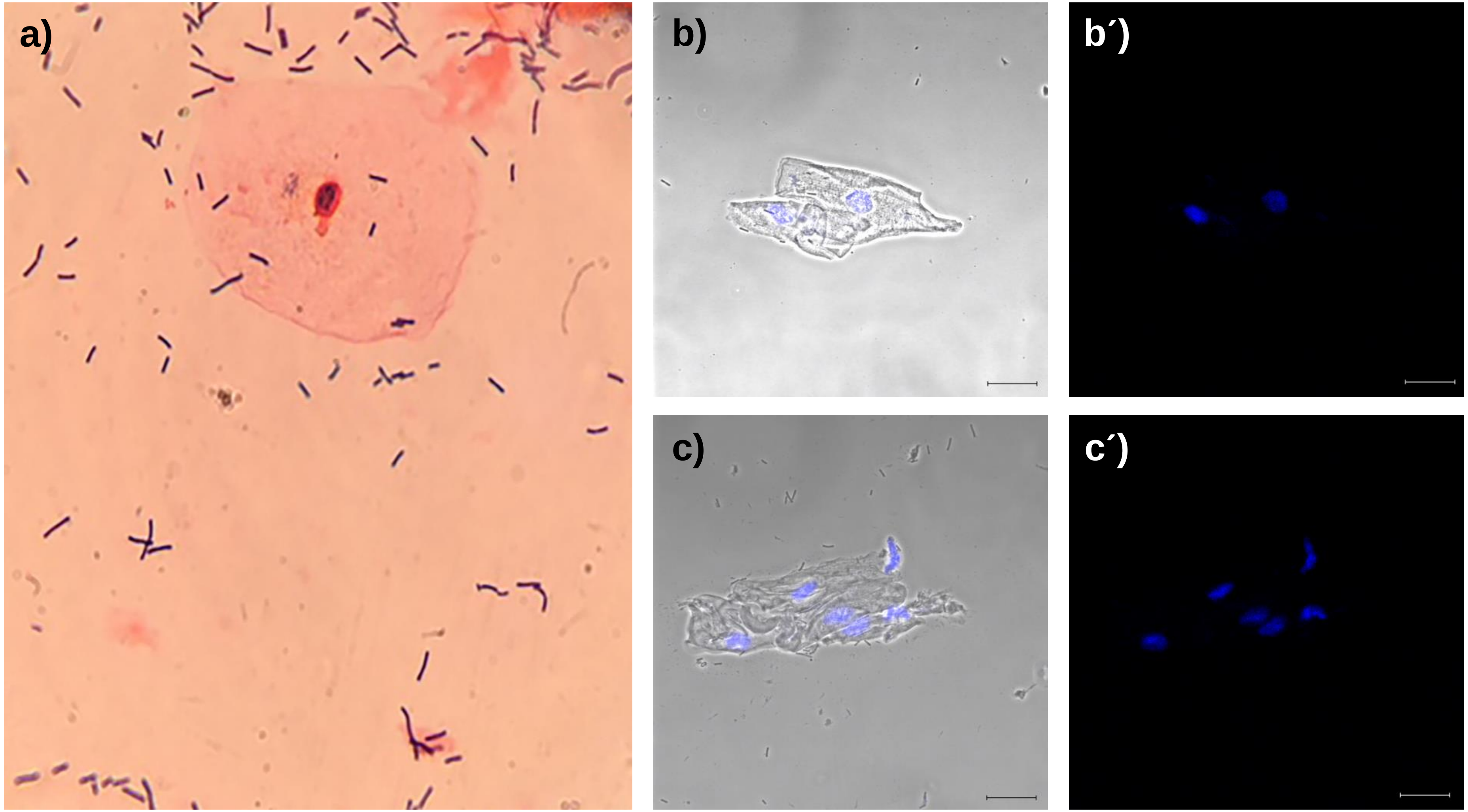

Fig. 1s. Sample from a clinically healthy donor without infection or inflammation. The donor 51 (from group 1) did not have symptoms, signs or diagnosis of infection or inflammation. **a)** Gram staining showed normal microbiota; **b- b')** Negative staining of *T. vaginalis* with specific antibodies α-*T. vaginalis;* **c- c´)** Epithelial cell nuclei were observed with Hoechst 33342 dye; LL-37 immunodetection was negative. Bar 20 μm.

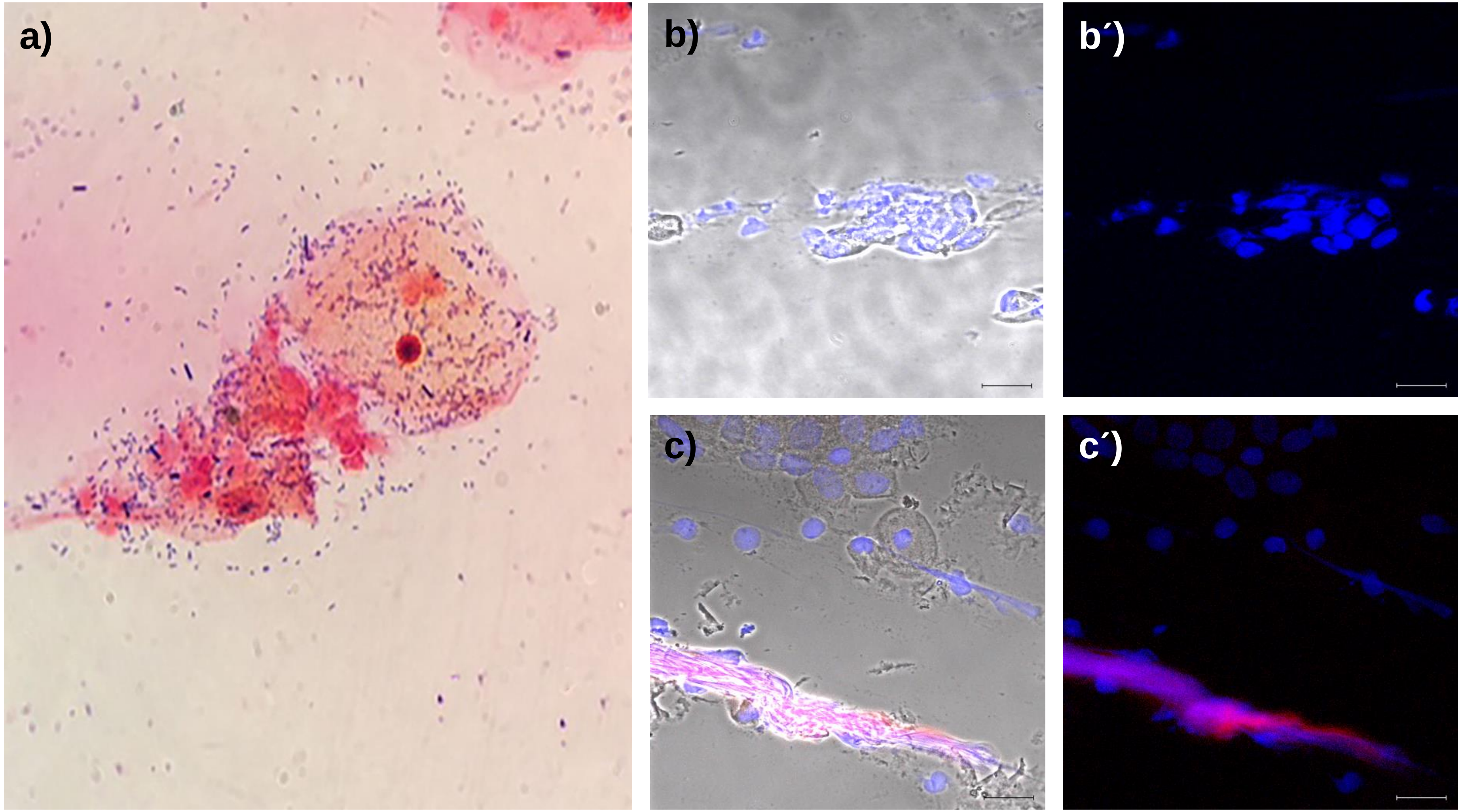

Fig. 2s. Sample from an asymptomatic donor diagnosed with bacterial vaginosis. Sample from an asymptomatic donor who arrived at the hospital for a routine Pap smear and was diagnosed with bacterial vaginosis. **a)** Staining showed few Gram-positive bacilli (normal microbiota) and abundant Gram-positive cocci, further identified as *Staphylococcus aureus*. **b)** *T. vaginalis* was negative; **c)** We also observed multilobed nuclei of neutrophils, epithelial cells and extracellular DNA associated with LL-37. Bar 20 μm.

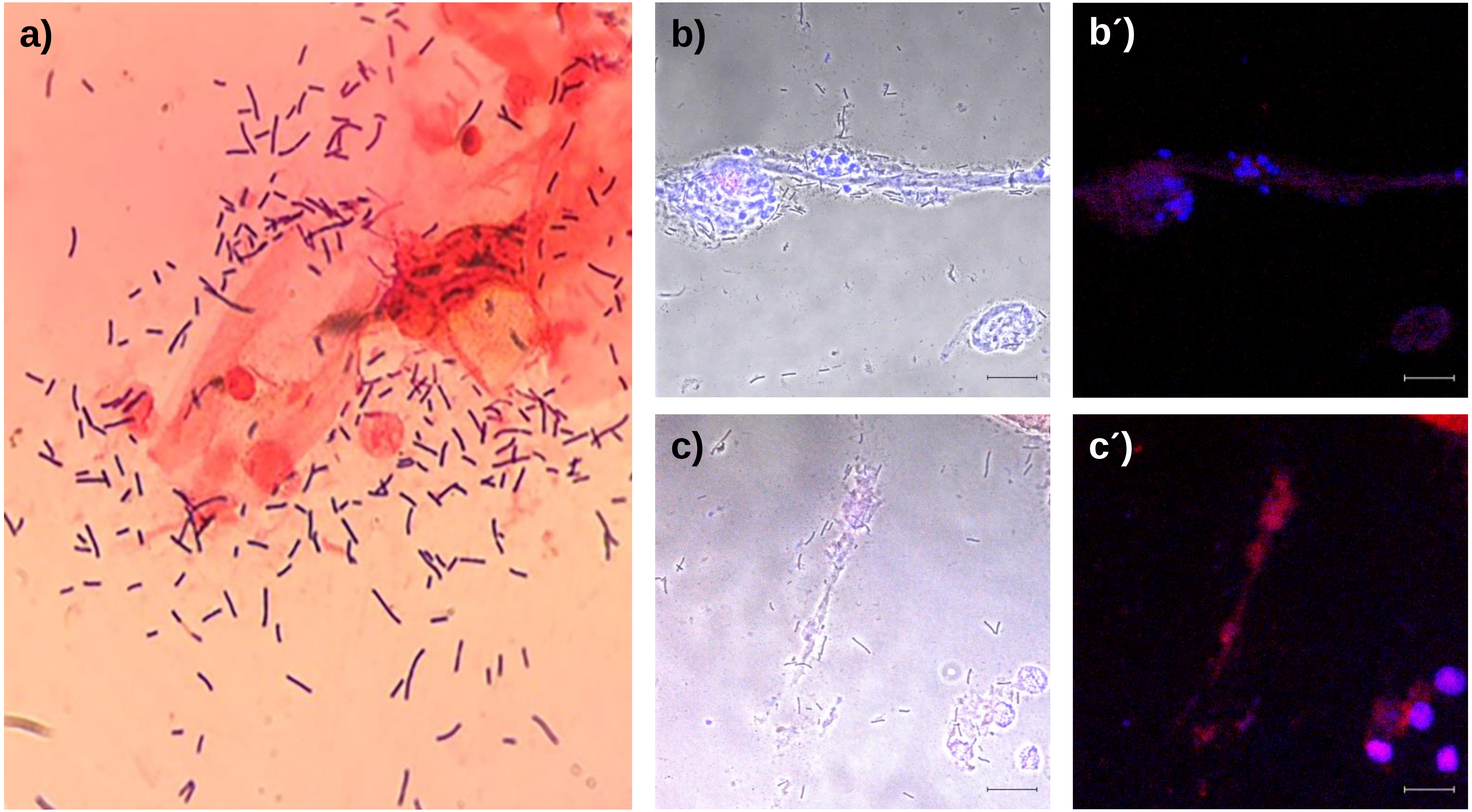

Figure 3s. Sample from an asymptomatic donor with trichomoniasis. The asymptomatic donor number 48 was diagnosed with trichomoniasis. **a)** Gram staining showed Gram-positive bacilli corresponding to the normal microbiota. **b- b')** *T. vaginalis* (red) was detected using specific antibodies and a secondary antibody coupled to Alexa Fluor 594; **c- c´)** Multilobed nuclei, DNA networks (blue) and LL-37 (red) were detected associated with the observed DNA networks. Bar 20 µm.

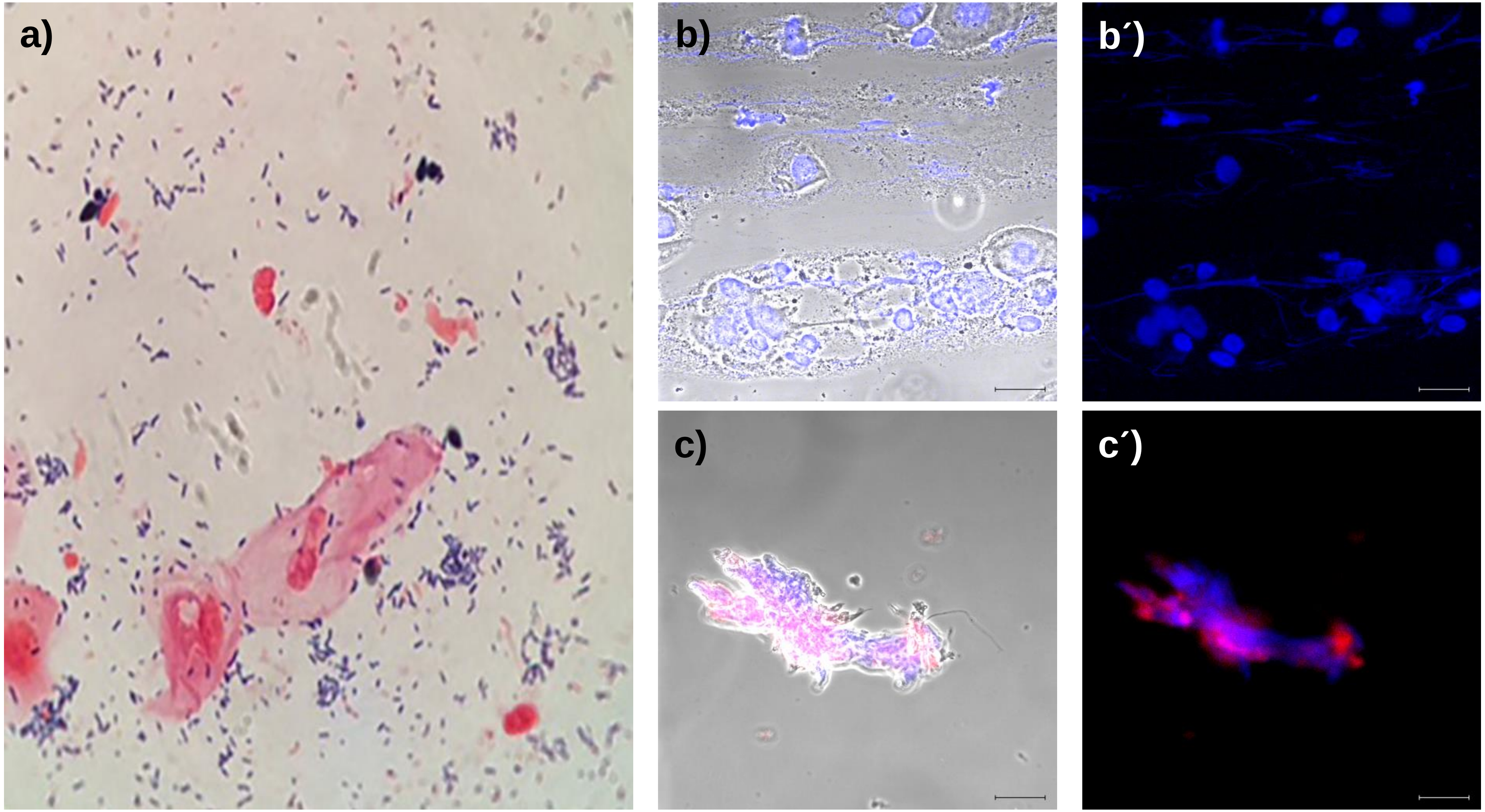

Figure 4s. Sample from an asymptomatic donor with candidiasis. The asymptomatic donor number 49 was diagnosed with candidiasis. **a)** Gram staining showed normal microbiota and yeasts; **b-b')** *T. vaginalis* was absent; **c- c´)** Extracellular DNA networks were stained with Hoechst 33342 (blue) and LL-37 immunodetected (red). Bar 20 µm.
